# Genetic Control of Ascorbic Acid Biosynthesis and Recycling in Horticultural Crops

**DOI:** 10.3389/fchem.2017.00050

**Published:** 2017-07-11

**Authors:** Ifigeneia Mellidou, Angelos K. Kanellis

**Affiliations:** ^1^Group of Biotechnology of Pharmaceutical Plants, Laboratory of Pharmacognosy, Department of Pharmaceutical Sciences, Aristotle University of Thessaloniki Thessaloniki, Greece; ^2^Laboratory of Agricultural Chemistry, Department of Crop Science, School of Agriculture, Aristotle University of Thessaloniki Thessaloniki, Greece

**Keywords:** ascorbate biosynthesis, GGP, ascorbate recycling, translation, transcription, vitamin C

## Abstract

Ascorbic acid (AsA) is an essential compound present in almost all living organisms that has important functions in several aspects of plant growth and development, hormone signaling, as well as stress defense networks. In recent years, the genetic regulation of AsA metabolic pathways has received much attention due to its beneficial role in human diet. Despite the great variability within species, genotypes, tissues and developmental stages, AsA accumulation is considered to be controlled by the fine orchestration of net biosynthesis, recycling, degradation/oxidation, and/or intercellular and intracellular transport. To date, several structural genes from the AsA metabolic pathways and transcription factors are considered to significantly affect AsA in plant tissues, either at the level of activity, transcription or translation via feedback inhibition. Yet, all the emerging studies support the notion that the steps proceeding through GDP-_L_-galactose phosphorylase and to a lesser extent through GDP-_D_-mannose-3,5-epimerase are control points in governing AsA pool size in several species. In this mini review, we discuss the current consensus of the genetic regulation of AsA biosynthesis and recycling, with a focus on horticultural crops. The aspects of AsA degradation and transport are not discussed herein. Novel insights of how this multifaceted trait is regulated are critical to prioritize candidate genes for follow-up studies toward improving the nutritional value of fruits and vegetables.

## Introduction

Ascorbic acid (AsA) or vitamin C is one of the most abundant water-soluble low molecular weight antioxidants found throughout the plant cells including the apoplast exerting a central role in regulating the cellular redox potential (Sanmartin et al., 2003; Fotopoulos et al., 2006, 2008; Foyer and Noctor, 2011; Fotopoulos and Kanellis, 2013; Gest et al., 2013). As humans and some other primates lack the ability to synthesize and store AsA, they depend on fresh fruits and vegetables to cover their daily requirements. All recent studies build a strong case toward a diet rich in AsA for improving human health (Troesch et al., 2012), suggesting that AsA should be a clear target for the nutritional enhancement of horticultural crops. Due to its remarkable functions in plant growth and development, as well as its benefits in human diet, AsA regulation in plant edible organs has received much attention in recent years.

The accumulation of AsA within the same species may vary between different cultivars (Bulley et al., 2009; Mellidou et al., 2012a,b; Gest et al., 2013), tissue types (Bulley et al., 2009), and developmental stages (Pateraki et al., 2004; Bulley et al., 2009; Ioannidi et al., 2009; Mellidou et al., 2012b). In spite of this variability, AsA is tightly regulated by the fine orchestration of net biosynthesis, recycling, degradation/oxidation, and/or intercellular and intracellular transport. Several transgenic approaches have been employed to enhance AsA accumulation in plants, involving primarily leaf material of model plants (*Arabidopsis* or tobacco). Nevertheless, overexpression of several structural AsA-related genes from various AsA metabolic pathways had so far limited success in most species, raising concerns on the significance of single structural genes in the control of AsA concentrations. In this regard, it may be required to interfere into entire regulatory networks using more than one structural genes or transcription factors (TFs) in order to enrich AsA levels beyond the current levels. Although numerous studies on genetic factors indicated that AsA accumulation showed a relatively high heritability (Davey et al., 2006; Mellidou et al., 2012a; Bulley and Laing, 2016), the expression of certain transcripts critical to AsA metabolic pathways has also been shown to respond to environmental stimuli such as alterations in light density, temperature, ethylene, low oxygen and wounding (Sanmartin et al., 2007; Yabuta et al., 2007; Ioannidi et al., 2009; Massot et al., 2012; Yoshimura et al., 2014). On the basis of these considerations, this mini review shall attempt to illustrate the genetic factors governing the AsA pool in plant tissues with a focus on horticultural crops.

## Genetic regulation through promoting biosynthesis

**Figure 1:**
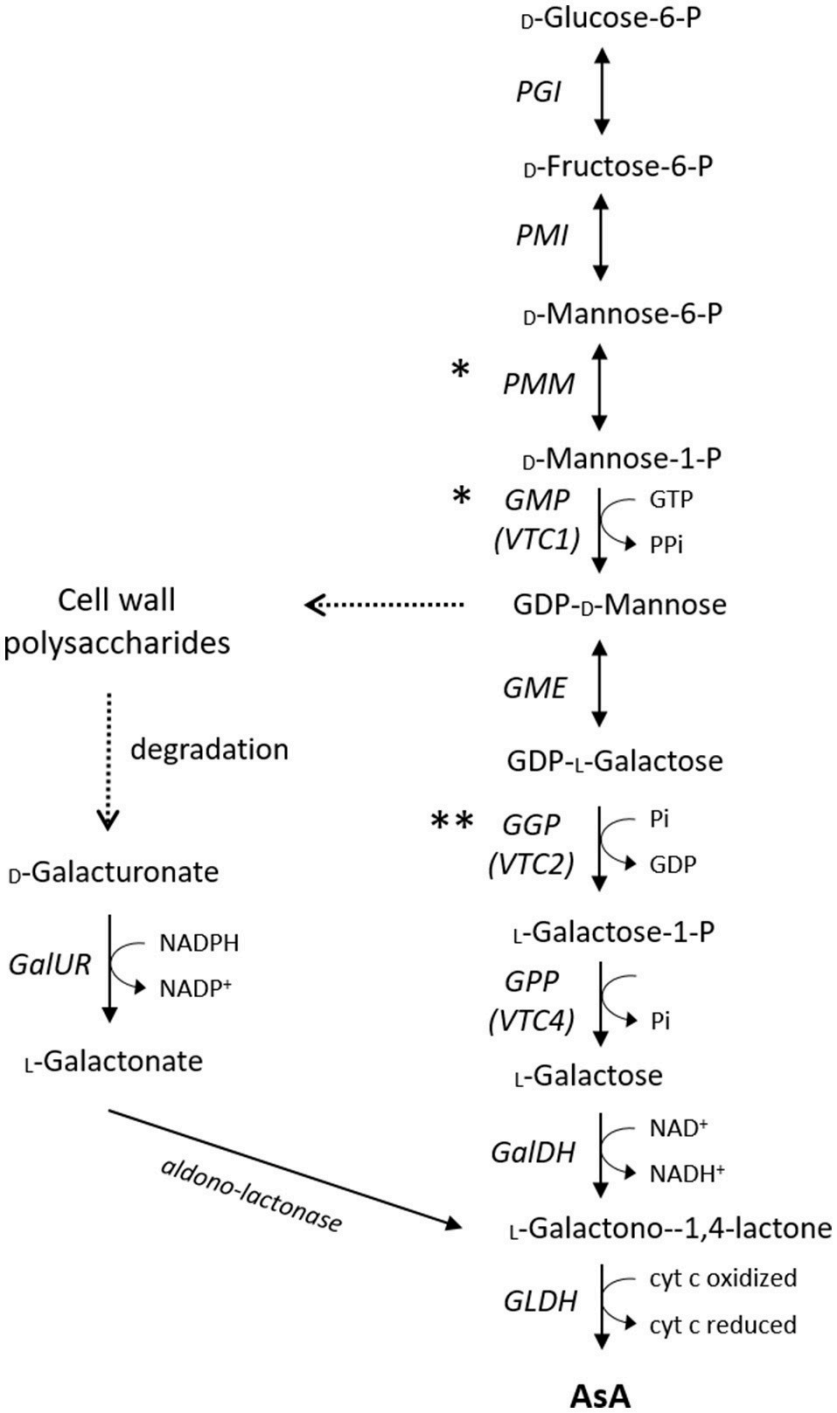
Major AsA biosynthetic pathways in plants. Asterisks indicate how transcription factors may influence AsA biosynthesis other than regulating gene transcription (^*^enzyme activity, ^**^ translation). The cut arrows indicate simplified reactions with missing steps. *PGI*, Phosphoglucose Isomerase; *PMI*, Mannose-6-phosphate isomerase; *PMM*, Phosphomannomutase; *GMP*, GDP-_*D*_-mannose pyrophosphorylase; GME, GDP-_D_-mannose 3′ 5′ epimerase; *GGP*, GDP-_L_-galactose-phosphorylase; *GPP*, _L_-galactose-1-P phosphatase; *GalDH*, _L_-galactose dehydrogenase; *GLDH*, _L_-galactono-1,4-lactone dehydrogenase; GalUR, _D_-galacturonate reductase.

In higher plants, the current consensus is that AsA biosynthesis from glucose via the so-called _L_-galactose pathway (Figure 1; Wheeler et al., 1998, 2015) is the dominant route for AsA accumulation. Although conclusive evidence for all the intermediate steps has only relatively recently become available with the fully characterization of the *Arabidopsis* AsA-deficient mutants (Conklin et al., 2006; Laing et al., 2007), several structural genes from this pathway have been proposed to be key regulators of AsA concentrations in various species. A plethora of successful and less successful biotechnological approaches has been employed to enhance the AsA pool size, overcoming specific rate-limiting steps of the _L_-galactose pathway. The early, non-specific for AsA synthesis, genes of the main pathway, such as phosphomannose isomerase (*PMI*) and phosphomanno mutase (*PMM*), are less possible to exert a major control over AsA homeostasis (Qian et al., 2007; Maruta et al., 2008). The role of the next two genes of the _L_-galactose pathway, named GDP-_D_-mannose pyrophosphorylase (*VTC1* or *GMP*) and GDP-_D_-mannose-3,5-epimerase (*GME*), is highly controversial. In the first case, expression of *GMP* has been correlated with AsA concentrations in some species, including acerola (Badejo et al., 2008), but not in tomato (Ioannidi et al., 2009), kiwifruit (Bulley et al., 2009), or blueberry (Liu et al., 2015). As *GMP* is also involved in cell wall polysaccharides synthesis and glycoproteins (Smirnoff, 2000), genes upstream this step are not solely committed to AsA biosynthesis. In the second case, a good correlation between *GME* transcripts and AsA had been reported in apple (Li et al., 2010b) and blueberry (Liu et al., 2015), but not in peach (Imai et al., 2009), tomato (Ioannidi et al., 2009; Mellidou et al., 2012b), or kiwifruit (Bulley et al., 2009). Modification of *GME* expression had only little effect on AsA pool of either leaf or fruit tissues (Bulley et al., 2009; Gilbert et al., 2009; Zhang et al., 2010; Mounet-Gilbert et al., 2016). Despite the fact that *GME* is not the rate-limiting step in AsA biosynthesis, it represents the intersection between the _L_-galactose pathway for the synthesis of AsA and the generation of monomers for cell wall biosynthesis, and it further seem to have distinct roles in pollen development, seed production, and vegetative growth (Mounet-Gilbert et al., 2016).

As GME catalyzes the double epimerization between _L_-galactose and _L_-gulose, a side branch from the main route has been reported to operate independently in terms of both enzyme specificity and subcellular location (Wolucka and Van Montagu, 2003), although its relevant contribution to AsA biosynthesis remains unknown. Beyond the ethical concerns for both humans and the environment, overexpression of the rat _L_-gulonolactone oxidase (*GULO*), the terminal enzyme of this “gulose shunt” significantly increased the AsA levels of several species (Jain and Nessler, 2000; Lim et al., 2012). Recently, *GULO* has been found to be functionally replaced with _*L*_-galactono-1,4-lactone dehydrogenase (*GLDH*) in photosynthetic eukaryotes in order to uncouple AsA biosynthesis from the generation of hydrogen peroxide with the acquisition of plastids through evolution (Wheeler et al., 2015).

Concerning the first committed step of the pathway, catalyzed by GDP-_L_-galactose phosphorylase (*GGP*), early studies on the ozone-sensitive AsA-deficient *vtc2 Arabidopsis* mutants (Conklin et al., 2000) showed that these mutants only contain 10–20% of the wild-type AsA levels resulting in insignificantly reduced growth. Recently, the presence of an independent cryptic mutation in *GGP* caused a similar reduction in AsA levels accompanied, however, by a smaller decrease in plant growth (Lim B. et al., 2016), pointing to a re-evaluation of *GGP* function in plants. Yet, several lines of evidence suggest *GGP* as the control point in the AsA biosynthetic pathway in several species including *Arabidopsis* (Bulley et al., 2009; Yoshimura et al., 2014), tobacco (Bulley et al., 2009; Wang et al., 2014), tomato (Bulley et al., 2012; Mellidou et al., 2012b; Wang L.-Y. et al., 2013), kiwifruit (Li et al., 2010c), citrus (Alós et al., 2013), blueberry (Liu et al., 2015), strawberry and potato tubers (Bulley et al., 2012). To a certain extent, this is fairly expected as *GGP* is placed at the first committed step of the pathway (Bulley and Laing, 2016). Further support to the pivotal role of *GGP* in AsA regulation arises from the fact that only *GGP* transcript levels significantly correlated with changes in AsA levels between high-AsA and low-AsA species in tomato (Mellidou et al., 2012b) and kiwifruit (Li et al., 2014). QTL studies in apple also provided strong evidence that *GGP* is the only structural gene tightly linked to flesh AsA levels independently of the environmental conditions (Mellidou et al., 2012a). Transient transformation using the kiwi or *Arabidopsis* genes revealed that *GGP* and *GME* operates synergistically to govern the AsA pool size (Bulley et al., 2009, 2012; Laing et al., 2015). Notably, when the transcript levels of either *GGP* or *GME* are modified (Gilbert et al., 2009; Zhang et al., 2010; Wang L.-Y. et al., 2013), the expression of the other one is also changed presumably to keep the balance in AsA biosynthesis and to maintain a stable AsA pool. Transcript levels of both genes are tightly regulated by light showing a diurnal trend (Dowdle et al., 2007; Massot et al., 2012). Specifically for *GGP*, its expression is culminated in the morning to support biosynthesis later on when maximum light density demands higher AsA levels (Dowdle et al., 2007). Allelic associations studies in apple reinforce the notion that SNPs found in *GGP* coding sequence are rather linked to polymorphisms in the promoter region that alter allele expression, than to altered protein function (Mellidou et al., 2012a).

Although the transcriptional control of *GGP* in regulating AsA accumulation is well recorded, it is only recently that its translational regulation has been elucidated under challenging conditions (Laing et al., 2015). The 5′-untranslated region (UTR) of *GGP* appears to contain a novel, highly conserved, non-canonical upstream open reading frame (uORF) in a wide range of species. Based on this observation, a model that allows feedback responsive regulation of AsA synthesis under unfavorable conditions has been proposed. According to this model, the uORF is translated and inhibits *GGP* translation at high AsA levels, while the uORF is disabled and *GGP* is translated at low AsA levels. The involvement of uORF in the regulation of *GGP* translation under rapidly altering conditions, without the need of gene transcription modification points out to a more robust way to control AsA concentrations. However, what merits further investigation is whether this conserved uORF and mutations within are able to explain the increased AsA levels found in some species or whether there are other factors synergistically or separately regulate *GGP* translation.

Despite the fact that expression patterns of the next gene of the pathway, namely _L_-galactose-1-P phosphatase (*GPP* or *VTC4*), were found to correlate with AsA concentrations in tomato and apple fruit (Ioannidi et al., 2009; Li et al., 2010b), in response to light (Yabuta et al., 2007; Yoshimura et al., 2014), ethylene, wounding, cold and post-anoxic conditions (Ioannidi et al., 2009), it seems that this step is not the rate-limiting factor in AsA biosynthesis (Conklin et al., 2006; Torabinejad et al., 2009; Mellidou et al., 2012b; Li et al., 2017). Regarding the other genes downstream this step [_L_-galactose dehydrogenase (*GalDH*) and _L_-galactono-1,4-lactone dehydrogenase (*GLDH*)], none of them were found to exert a significant effect on AsA pool, at least in tomato fruit (Alhagdow et al., 2007; Mellidou et al., 2012b). Recently however, *GLDH* has been proposed as a key step in the regulation of AsA accumulation in pepper, being probably involved in the transport of AsA among different organs (Rodríguez-Ruiz et al., 2017).

The most extensively studied alternative route of AsA synthesis is the one through _D_-galacturonic acid (Figure 1), which is used for the synthesis of _L_-galactonic acid derivatives via _D_-galacturonate reductase (*GalUR*) (Agius et al., 2003; Badejo et al., 2012). Therefore,_D_-galacturonic acid has a dual role, one being as a key component of cell wall pectins, the other being as a substrate for AsA biosynthesis. Several lines of evidence suggest that AsA synthesis via this pathway may occur in certain species such as strawberry (Agius et al., 2003), orange (Xu et al., 2012), apple (Mellidou et al., 2012a), grape (Cruz-Rus et al., 2010), and rose (Li et al., 2017), or at specific developmental stages i.e., ripe tomato fruit (Badejo et al., 2012). Additionally, increasing AsA levels in tomato plants via expressing the strawberry *GalUR* has led to enhanced resistance to various abiotic stress factors (Lim M. Y. et al., 2016). Apart from UDP-glucose, the glucoronate pathway for the synthesis of AsA can be also derived from *myo*-inositol (MI). However, published data suggest that this pathway is primarily involved in hexose metabolism, as well as in starch and cell wall pectin biosynthesis, rather than in AsA biosynthesis, in which MI is quite unlikely to serve as an AsA precursor (Endres and Tenhaken, 2009; Mellidou et al., 2012b). Nevertheless, as this alternative pathway is considerably shorter than the _*L*_-galactose pathway, it may complement the predominant biosynthetic route particularly in fruit tissues under stress conditions (Cruz-Rus et al., 2011).

## Genetic regulation through enhancing recycling

As an antioxidant, AsA is able to accept electrons from a wide range of free radicals and in this process it undergoes enzymatic regeneration from its oxidized forms, monodehydroascorbate (MDHA) and dehydroascorbate (DHA). Thereafter, these oxidized forms of AsA can be regenerated by the so-called ascorbate-glutathione (GSH) cycle, so that GSH and the activities of GSH reductase (GR), dehydroascorbate reductase (DHAR), and monodehydroascorbate reductase (MDHAR) preserve AsA homeostasis (Foyer and Noctor, 2011). Overall, the current consensus is that increased AsA levels through enhanced recycling could provide a greater direct protection against free radicals than through increased biosynthesis.

The role of *MDHAR* in governing AsA pool size has been clearly demonstrated in tomatoes using either QTL mapping and introgression lines (Stevens et al., 2007, 2008; Sauvage et al., 2014), or through assessing expression and activity profiles throughout ripening (Mellidou et al., 2012b). Furthermore, *MDHAR* expression has been correlated with AsA accumulation in blueberry (Liu et al., 2015). Overexpression of *MDHAR* had no particular effect on AsA pool in tobacco (Yin et al., 2010), and tomato fruit (Haroldsen et al., 2011), or even exerts a negative effect over the AsA pool (Gest et al., 2013). The unexpected effect of this *MDHAR* on AsA levels cannot be justified by alterations in the expression of biosynthetic genes, or the activity of recycling enzymes. The authors hypothesized that changes in MDHAR activity in the transgenic lines may trigger a signal that stimulates changes in AsA content and the redox state through an unidentified mechanism of interactions/compensations (Gest et al., 2013). Suppression of *MDHAR* has led to slight diminished build-up of AsA degradation product in tomatoes, indicating that controlling the recycling rate via reducing MDHAR activity can be an efficient solution toward enhancing the protection of AsA pool (Truffault et al., 2017). Additionally, reduced MDHAR activity via RNAi strategies decreased tolerance to cold storage and affected AsA levels to some extent in tomatoes (El Airaj et al., 2013). To the contrary, overexpression of a chloroplastic *MDHAR* in tomato led to enhanced AsA concentration (Li et al., 2010a), highlighting the different function that the organelle-specific isoforms exert over the AsA pool.

On the other hand, QTL studies in apple fruit revealed the involvement of *DHAR in* regulating the redox state of the AsA pool; further, increased flesh DHA concentrations were associated with susceptibility to postharvest disorders such as flesh browning (Mellidou et al., 2012a). Transcriptomic studies uncovered that prolonged postharvest storage downregulated *DHAR* expression resulting in the irreversibly oxidation of AsA and thus enabling browning to occur (Mellidou et al., 2014). Furthermore, *DHAR* expression has been correlated with AsA accumulation in chestnut rose (Huang et al., 2014), and in blueberry (Liu et al., 2015). Overexpression of *DHAR* has been evaluated in several species, and resulted in a remarkable increase of AsA pool in *Arabidopsis* leaves (Wang et al., 2010), in maize kernels and leaves (Chen et al., 2003; Naqvi et al., 2009), in rice (Kim et al., 2013), as well as in tobacco leaves (Chen et al., 2003; Eltayeb et al., 2006), but not in potato leaves and tubers (Qin et al., 2010). Despite the fact that the initial attempts to enhance AsA recycling yielded no astonishing results, yet the potential value of increasing the efficiency of AsA regeneration should be further studied, mainly because a number of QTLs were linked to *DHAR* and *MDHAR*.

## Genetic regulation through hormones and other proteins/factors

Recent studies implicate the involvement of hormone interactions in regulating AsA accumulation, a notion previously underestimated. AsA is known to be involved in the synthesis of ethylene as a co-factor of 1-aminocyclopropane-1-carboxylate (ACC) oxidase to convert ACC to ethylene, having thus major roles in fruit development and ripening. In this regard, ACC oxidase is negatively correlated with AsA content in tomatoes (Lima-Silva et al., 2012). Apart from ethylene synthesis, ethylene signaling seems to be involved in AsA accumulation. The increased availability of intermediates for the main biosynthetic pathway due to cell wall pectin degradation triggered by ethylene can increase the flux toward AsA synthesis (Figure 1). Furthermore, the tomato mutants *Never-ripe* that fail to ripe normally, have high AsA content (Osorio et al., 2012), suggesting a possible negative link between AsA accumulation and ethylene perception that needs to be further explored. Several other genes involved in hormone homeostasis and signaling have been proposed to significantly correlate with AsA levels either positively including the protein phosphatase 2C, which is a negative regulator of abscisic acid (ABA) responses, and gibberellin oxidases, or negatively such as a green ripe-like and a brasinoesteroid-regulated xyloglucan endo-transglycosylase (Lima-Silva et al., 2012). Similarly, co-expression of *Stylosanthes guianensis* (stylo) 9-cis-epoxycarotenoid dioxygenase and of yeast _D_-arabinono-1,4-lactone oxidase resulted in increased AsA levels and elevated tolerance to both drought and chilling stress (Bao et al., 2016).

**Figure 2:**
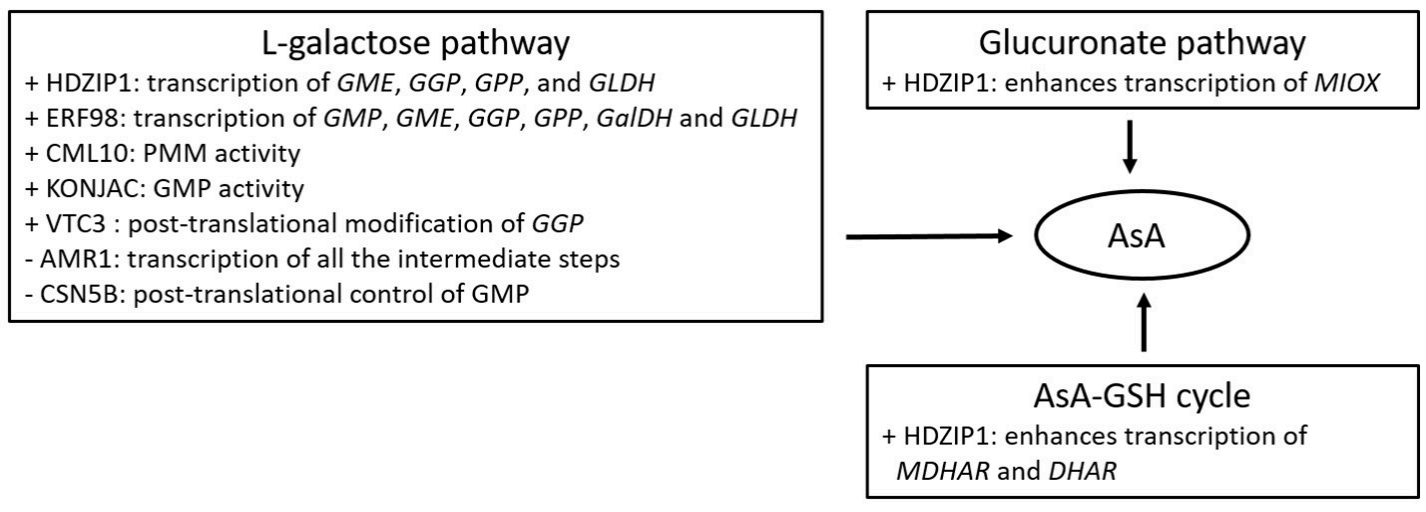
Summary of proteins/factors affecting either positively (+), or negatively (−) the AsA metabolic pathways.

Several TFs have also been proposed to govern AsA levels in plants exposed to oxidative stress or during plant growth (Figure 2). Some of them may exert a positive effect over the AsA pool size through enhancing transcription of the biosynthetic genes such as the ethylene response factor ERF98 (Zhang et al., 2012) in *Arabidopsis*, or the HD-ZIP I (Hu et al., 2016) and the auxin/indolo acetic acid (Aux/IAA) TF in tomato (Lima-Silva et al., 2012). Others may exert a negative impact on AsA concentrations such as the ERF33 in tomato (Lima-Silva et al., 2012), or may regulate AsA-dependent growth such as the ABSCISIC ACID-INSENSITIVE-4 TF (ABI4; Kerchev et al., 2011) in *Arabidopsis*. Similar results have been recorded in ripening tomato fruit, with several TFs including MYB, NAC and ZIF, modulating transcript levels of AsA biosynthetic genes, and correlating with the accumulation of AsA (Ye et al., 2015). Nevertheless, overexpression or reducing the expression of ERF98 and HD-ZIP I showed a moderate effect on the AsA concentration (Bulley and Laing, 2016), questioning their significance in governing AsA pool. In addition, some TFs significantly correlated with DHA accumulation, rather than total AsA abundance (Ye et al., 2015), indicating the potential linkage between TFs and redox homeostasis.

There are also factors affecting enzyme or TF concentrations, such as VTC3 (Conklin et al., 2013), a component of the photomorphogenic COP9 signalosome (CSN5B; Wang J. et al., 2013), and AMR1 (AsA mannose pathway regulator 1; Zhang et al., 2009), all identified in *Arabidopsis* (Figure 2). VTC3, the last AsA-deficient *Arabidopsis* mutant loci with unknown function, has been predicted to encode a novel highly conserved dual function protein kinase/protein phosphatase 2C (Conklin et al., 2013). With two signal transduction domains, VTC3 may be involved in signal transduction regulating AsA levels in response to adverse environments. Furthermore, as its transcripts are constantly expressed across a wide range of conditions, its control over AsA pool may occur at the posttranslational level. Bulley and Laing (2016) proposed that VTC3 may be implicated in regulating the noncanonical highly conserved uORF of *GGP*. On the other hand, CSN5B and AMR1 exert a negative role over AsA levels in that a decrease in their transcript levels enhance AsA accumulation. Interestingly, a decrease in the expression of *AMR1* led to a 2-fold increase of AsA concentrations, as well as a boost in transcript levels of *GGP, GME* and *GMP* (Zhang et al., 2009). On the other hand, CSN5B was also found to interact with GMP affecting the light-dark control of AsA biosynthesis, indicating that CSN5B may be a potential posttranslational regulator of AsA synthesis (Wang J. et al., 2013; Bulley and Laing, 2016). A point mutation of VTC1 impairs the interaction with CSN5B and results in increased AsA biosynthesis and *Arabidopsis* seedling growth (Li et al., 2016). Other factors including nucleotide sugar pyrophosphorylase-like proteins (KONJAC1 and 2; Sawake et al., 2015), and a calmodulin-like protein (CML10; Cho et al., 2016), have been found to stimulate enzyme activity of AsA-related genes, revealing novel mechanisms of how the AsA pool is regulated. Specifically, KONJAC1 and 2 can stimulate GMP activity, while CML10 the activity of PMM resulting in increased cellular levels of mannose-1-phospate and consequently the subsequent reactions in the AsA biosynthetic pathway. Reducing gene expression of KONJACs and CML10 led to a 0.4 and 0.5–0.75-fold change in AsA concentrations, respectively (Bulley and Laing, 2016). As all previous reports have been conducted on model plant species, it remains an open question whether these factors operate similarly in horticultural crops.

## Conclusion and perspective

Emerging evidence provides important clues on the mechanism by which *GGP*, the key AsA biosynthetic gene, regulates the AsA pool size in several species, either through variations in *GGP* transcript levels or through feedback control at the translational level. Recent advances clearly suggest that inducing or inhibiting *GGP* translation via a highly conserved uORF may serve as a prominent mechanism to control the AsA accumulation rapidly under unfavorable environmental conditions. On the contrary, plethora of studies reinforce the hypothesis that induction of the other genes of the L-galactose pathway may be important in certain species, tissues, or developmental stages and may be responsible for the tissue acclimation under slowly changing conditions, or at certain environmental/developmental conditions. Further, it seems that the AsA pool can be also regulated via other metabolic pathways than biosynthesis and recycling that could affect either substrate availability, or control the expression levels of AsA-related genes at either the transcriptional or posttranslational level. However, recent advances on genetic engineering using either AsA structural genes or TFs and other factors clearly suggest that improving AsA concentrations beyond certain thresholds is harder than initially believed. Therefore, efforts should be directed toward understanding whether structural genes and TFs act individually or collectively to govern AsA accumulation, as well as what is the effect of environment on these systems in horticultural crops rather than model species. It also remains an open question why wild accessions tend to contain higher AsA levels than modern cultivars (up to 5-fold in tomato), or what is the source of variation within species (up to 10-fold in kiwifruit), and which genes/factors govern these mechanisms.

## Author contributions

IM organized and drafted this manuscript and AKK contributed to the editing of the manuscript. All authors read and approved the manuscript.

### Conflict of interest statement

The authors declare that the research was conducted in the absence of any commercial or financial relationships that could be construed as a potential conflict of interest.
